# Targeting a ribonucleoprotein complex containing the caprin-1 protein and the c-Myc mRNA suppresses tumor growth in mice: an identification of a novel oncotarget

**DOI:** 10.18632/oncotarget.3236

**Published:** 2014-12-10

**Authors:** Ya-Qi Qiu, Cheng-Wei Yang, Yue-Zhi Lee, Ruey-Bing Yang, Chih-Hao Lee, Hsing-Yu Hsu, Chien-Chung Chang, Shiow-Ju Lee

**Affiliations:** ^1^ Institute of Biotechnology and Pharmaceutical Research, National Health Research Institutes, Miaoli, Taiwan; ^2^ Cellular Biology, National Tsing Hua University, Hsinchu, Taiwan; ^3^ Institute of Biomedical Sciences, Academia Sinica, Taipei, Taiwan; ^4^ Department of Genetics and Complex Diseases, Division of Biological Sciences, Harvard School of Public Health, Boston, Massachusetts, USA

**Keywords:** Caprin-1, c-Myc, Cyclin D, G3BP1, Processing Body

## Abstract

Tylophorine compounds have been the focus of drug development for decades. Tylophorine derivatives exhibit anti-cancer activities but their cellular targets remain unknown. We used a biotinylated tylophorine derivative to probe for the interacting cellular target(s) of tylophorine. Tylophorine directly binds to caprin-1 and consequently enhances the recruitment of G3BP1, c-Myc mRNA, and cyclin D2 mRNA to form a ribonucleoprotein complex. Subsequently, this tylophorine targeted ribonucleoprotein complex is sequestered to the polysomal fractions and the protein expressions of the associated mRNA-transcripts are repressed. Caprin-1 depleted carcinoma cells become more resistant to tylophorine, associated with decreased formation of the ribonucleoprotein complex targeted by tylophorine. Consequently, tylophorine downregulates c-Myc and cyclins D1/D2, causing hypophosphorylation of Rb and suppression of both processing-body formation and the Warburg effect. Gene expression profiling and gain-of-c-Myc-function experiments also revealed that the downregulated c-Myc contributes to the anti-oncogenic effects of tylophorine compounds. Furthermore, the potent tylophorine derivative dibenzoquinoline-33b elicited a similar effect, as c-Myc protein levels were also decreased in xenograft tumors treated with dibenzoquinoline-33b. Thus, tylophorine compounds exert anti-cancer activity predominantly by targeting and sequestering the caprin-1 protein and c-Myc mRNA associated ribonucleoprotein complex.

## INTRODUCTION

c-Myc is a transcriptional factor that regulates a variety of cellular processes including the activity of cyclin D-Cdk (cyclin-dependent kinase) [1], the Warburg effect in cancer cell metabolism [2, 3], and cellular mRNP processing [4, 5]. Myc expression is deregulated in a wide range of human cancers and is therefore a therapeutic target. Recently, it was found that the RNA-binding protein human antigen R inhibits c-Myc expression by recruiting miRNA let-7-loaded RISC (RNA miRNA-induced silencing complex) to the c-Myc 3′UTR [6]. This raises the possibility of developing c-Myc inhibitors by targeting its associated RNP.

Cyclin D (cyclin D1, cyclin D2, and cyclin D3 encoded in human cells) is a member of the cyclin protein family. These proteins are involved in the regulation of cell cycle progression for the transition of G1 to S phase [7]. c-Myc has been shown to regulate transcription of genes involved in cell cycle including cyclin D [1]. The retinoblastoma tumor suppressor protein (pRb) is one of the best known substrates of active cyclin D-Cdk4/6 complexes. Thus, the phosphorylated pRb is a common downstream effector of cyclins D1 [8, 9], D2 [10, 11], and c-Myc [1, 7].

The tumor suppressor protein pRb is dysregulated in several major cancers. pRb is a stable protein and is phosphorylated by several Cdks, which down-regulates its activity. Active pRb acts as a tumor suppressor by inhibiting cell cycle progression [7]. In the hypophosphorylated active form, pRb sequesters the transcriptional factor E2F. When pRb is inactivated by hyperphosphorylation, it releases E2F, permitting E2F to execute its cell cycle regulatory functions. Therefore, the hyperphosphorylation of pRb during late G1 phase enables the activation of E2F-dependent transcription, e.g cyclin A2 [12].

Caprin-1(Cytoplasmic activation- and proliferation-associated protein 1) is ubiquitously expressed, and its phosphorylation is required for normal cell cycle progression from the G1 to S phase. Caprin-1 co-localizes with G3BP1 (Ras GTPase-activating protein-binding protein 1) within the cytoplasmic RNA granules that are associated with microtubules. This caprin-1/G3BP1 complex is thought to regulate the transport and translation of mRNAs whose protein products are involved in proliferation and migration in multiple cell types because the carboxyl-terminal region of caprin-1 selectively binds to c-Myc or cyclin D2 mRNAs [13]. Collectively, the caprin-1, G3BP1, c-Myc mRNA, and Cyclin D2 mRNA complex represents a function-specific RNP complex and is required for important cellular events.

Natural products constitute a successful and promising resource for drug discovery. The elucidation of the cellular targets of therapeutic natural products not only contributes to the identification of valuable novel drug targets for future development, but also guides subsequent molecular pharmacology and mode-of-action studies [14, 16]. Tylophorine-related natural compounds, which are found in the Asclepiadaceae and Moraceae plant families, exhibit multiple biological activities (e.g., anti-cancer, anti-coronavirus, and anti-inflammatory activities) [17-24]. Tylophorine compounds have been the focus of drug development as therapeutic agents since the 1960s, whereas tylocrebrine failed phase I clinical trials due to neurotoxicity [17]. Although several modes of action for anti-cancer tylophorine compounds have been reported [20, 25-28], their direct cellular target(s) and related pathways remain to be elucidated [24]. In particular, the fundamental mechanisms underlying the broad spectrum, drug-resistant cancer cell inhibitory activities of tylophorine compounds remain to be investigated.

We designed and synthesized a biotinylated tylophorine compound which retained the molecular targeting moiety, enabling the probing of direct cellular target(s). In combination with proteomic approaches and further molecular mechanism studies, the anti-cancer activities of the tylophorine compounds were elucidated. Our results provide a mechanistic basis for developing the tylophorine derived compounds into anti-cancer drugs. The identified cellular target(s) of tylophorine represent novel drug targets for anti-cancer therapeutic applications.

## RESULTS

### Synthesis of a biotinylated tylophorine analogue

**Figure 1:**
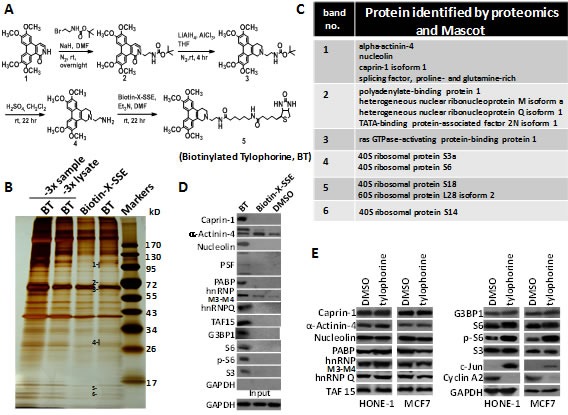
The application of biotinylated tylophorine to probe its direct interacting cellular targets in carcinoma cells A. Scheme for the synthesis of biotinylated tylophorine (BT), a dibenzoquinoline derivative. B & C. Pull-down experiment in HONE-1 cell lysates to identify potential molecular targets of tylophorine. BT and biotin-X-SSE were used to pull down the interacting molecules by using streptavidin beads. The lane labelled “3x sample” was loaded with 3-fold-amount of the BT pull-down and the lane labelled “3x lysate” was loaded with the BT pull-down from a 3-fold-amount of the lysate. The indicated bands in the silver-stained SDS-PAGE gel (B) were subjected to proteomic studies for Mascot analysis as potential targets (C). D. Immunoblot analyses with the indicated antibodies to confirm the molecules identified by Mascot analysis as potential tylophorine-interacting targets in the pulled-down complexes. E. Immunoblot analysis of the identified intracellular targets of biotinylated tylophorine in the total carcinoma cell lysates of HONE-1 or MCF7 cells treated with tylophorine for 24 h. The downregulation of cyclin A2 [20] and upregulation of c-Jun [25] protein expression induced by tylophorine treatment were considered as internal controls in addition to the loading control GAPDH. The results shown are representative of 3 independent experiments.

Previously, we reported that the non-planar structure between the two major phenanthrene and indolizidine/quinolizidine moieties, as well as the planarity and rigidity of the indolizidine/quinolizidine moiety of tylophorine compounds, significantly affect the activities of tylophorine analogues [19]. Recently, we successfully synthesized potent tylophorine-derived dibenzoquinolines that elicit the same biological functions as traditional pentacyclic tylophorine compounds [23]. Therefore, we concluded that the angular dibenzoquinoline moiety in tylophorine compounds is responsible for the interactions with their direct target molecule(s). Thus, we sought to design and synthesize a biotinylated tylophorine-derived dibenzoquinoline (Fig. 1A), referred to herein as the “biotinylated tylophorine”, to probe direct interacting molecules in cells. The biotinylated tylophorine retained anti-cancer cell activity with IC_50_ values of ~13-30 μM against HONE-1, MCF7, or NUGC-3 carcinoma cells (see Supplemental Table 1).

### Identification of direct targets of tylophorine via pull-down experiments with biotinylated tylophorine

HONE-1 cell lysates were incubated with the vehicle control (dimethyl sulfoxide, DMSO), biotin-X-SSE, or biotinylated tylophorine prior to the addition of streptavidin in a pull-down experiment. The pulled-down complex was subjected to sodium dodecyl sulfate-polyacrylamide gel electrophoresis (SDS-PAGE) and silver staining to analyze the proteins that specifically interacted with the biotinylated tylophorine. A total of 6 protein bands were specifically associated with biotinylated tylophorine compared to biotin-X-SSE (Fig. 1B); these bands were excised for further matrix-assisted laser desorption ionization-time of flight (MALDI-TOF) mass spectrometry (MS) proteomics analyses. The potential tylophorine-interacting proteins were identified by Mascot data analysis (Fig. 1C and Supplemental Table 2), and the specific interaction of these proteins with biotinylated tylophorine was validated by western blot analysis (Fig. 1D). Caprin-1, α-actinin-4, nucleolin, the splicing factor (PSF), polyadenylate-binding protein-1 (PABP-1), heterogeneous nuclear ribonucleoprotein M3-M4 (hnRNPM3-M4), hnRNPQ, TATA-binding protein-associated factor 2N isoform 1 (TAF-15), G3BP1, 40S ribosomal protein S6 (S6), phosphorylated S6 (p-S6), and ribosomal protein S3 were specifically pulled down by biotinylated tylophorine compared to biotin-X-SSE or DMSO, but the expression levels of these proteins in tylophorine-treated HONE-1 or MCF7 carcinoma cells were not significantly altered compared with DMSO-treated cells, with the exception of p-S6, which was significantly increased (Fig. 1E).

The pull-down of ribosome proteins by biotinylated tylophorine was not unexpected because tylocrebrine was previously reported to inhibit the translocation of peptidyl-tRNA in ribosomes by 54%, although a tylocrebrine concentration as high as 100 μM was used [29]. Because tylophorine compounds exert significant anti-cancer activities, generally with IC_50_ values ranging from hundreds to a few nM [22-24], we further examined other proteins identified to seek specific targets for anti-cancer.

### Tylophorine associates with an RNP complex containing caprin-1, G3BP-1, c-Myc mRNA, and Cyclin D2 mRNA

**Figure 2:**
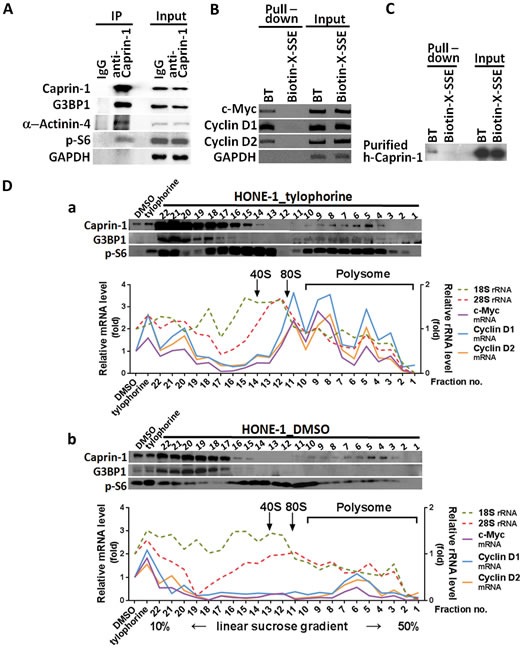
The association of caprin-1 and G3BP1 with c-Myc and cyclins D1/D2 mRNAs through tylophorine A. The co-immunoprecipitation of caprin-1 with α-actinin-4, G3BP1, and p-S6 from the HONE-1 cell lysate in the presence of biotinylated tylophorine was detected by immunoblot analyses with the indicated antibodies. B. Biotinylated tylophorine pulled down the mRNAs for c-Myc and cyclins D1 and D2 from the HONE-1 cell lysate as detected by RT-PCR with the indicated primer pairs. C. Purified recombinant caprin-1 protein physically associates with biotinylated tylophorine. Human caprin-1 was transiently expressed in HEK-293 cells, affinity-purified as described in the Methods section, and applied to a pull-down experiment with biotinylated tylophorine. The protein was subsequently detected by immunoblot analysis using an antibody against human caprin-1. D. Polysome profile analysis (by sucrose gradient sedimentation) and protein (by western, upper panel) and RNA (by qRT-PCR, lower panel) analyses of sucrose gradient fractions for the co-localization of caprin-1, G3BP1, c-Myc mRNA, cyclin D1 mRNA, and cyclin D2 mRNA in the tylophorine-induced RNP complex from the lysates of HONE-1 cells treated with either with tylophorine (2 μM) (Fig. 2D-a) or vehicle (0.1% DMSO) (Fig. 2D-b) for 24 h. BT, biotinylated tylophorine. The results shown are representative of 3 independent experiments.

Caprin-1 was identified among the molecules that were pulled down by biotinylated tylophorine, and we focused on this protein because it is ubiquitously expressed and required for normal progression through the G1-S phase [13]. We performed co-immunoprecipitation using a caprin-1 antibody to assess the associated complex members. We identified α-actinin-4, G3BP1, and p-S6 within the caprin-1 complex in the presence of biotinylated tylophorine (Fig. 2A). Because caprin-1 and G3BP1 were previously reported to associate and co-localize within RNA-rich cytoplasmic granules [13], we further dissected the association of c-Myc mRNA and cyclin D2 mRNA in HONE-1 cells by RT-PCR analysis of the products from the pull-down experiment with biotinylated tylophorine (Fig. 2B). We also demonstrated the direct, but weak, interaction of biotinylated tylophorine with caprin-1 in another pull-down experiment using purified recombinant caprin-1 protein (Fig. 2C).

**Figure 3:**
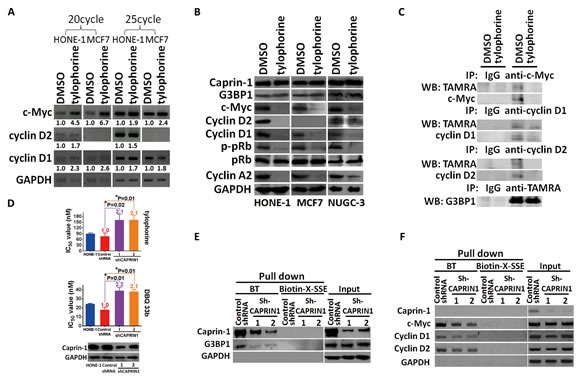
The effect of tylophorine through caprin-1 on c-Myc, cyclin D1, and cyclin D2 expression in carcinoma cells A. Semi-quantitative RT-PCR analyses of the effect of tylophorine on the mRNA levels of c-Myc, cyclin D1, and cyclin D2. The relative expression levels of each mRNA were normalized with their respective internal loading control GAPDH. B. Immunoblot analyses of the effects of tylophorine on protein expressions related to the caprin-1-associated RNP complex and their common downstream target pRb. The carcinoma cells were treated with tylophorine (2 μM) for 24 h prior to semi-quantitative RT-PCR or western blotting analyses with the indicated gene primer pairs or antibodies. C. Tylophorine repressed the de novo protein syntheses of c-Myc and cyclins D1/D2. TAMRA-labeled newly synthesized proteins from tylophorine treated HONE-1 cells were immunoprecipitated with specific antibody as indicated and then detected by western blotting with indicated antibody. D. Depletion of caprin-1 increased carcinoma cells' resistance to tylophorine or DBQ 33b treatments. HONE-1 cells were transfected with control shRNA or CAPRIN1 shRNA plasmids respectively, and then selected with puromycin. The resultant cells were analyzed by western blotting for validation of caprin-1 depletion before subjected to measurement of cell growth IC_50_ values by tylophorine or DBQ 33b. A 2-tailed unpaired Student's t test was used to evaluate the p-value between two groups. E & F. The effect of caprin-1 depletion on the association of tylophorine targeted RNP complex. Caprin-1 depleted HONE-1 lysates described in D were subjected to pull-down assays with biotinylated tylophorine or biotin-X-SSE. Immunoblot (E) and Semi-RT-qPCR analyses (F) were used to detect the protein and mRNA components in the tylophorine-associated RNP complex. The sequences of the gene primer pairs used are listed in Supplemental Table 3. BT, biotinylated tylophorine. The results shown are representative of 3 independent experiments. *, p<0.05.

Furthermore, sedimentation fractionation revealed that caprin-1, G3BP1, p-S6, c-Myc mRNA, and cyclin D2 mRNA co-localized within polysomal fractions upon tylophorine treatment (Fig. 2D-a); this colocalization did not occur in vehicle-treated HONE-1 cells (Fig. 2D-b). Moreover, a low abundance of cyclin D2 has been observed in certain carcinoma cell lines [30-32], such as MCF7 (Fig. 3A); under certain conditions, the loss of a specific cyclin can be compensated by the presence of others [33, 34]. Further investigation revealed that cyclin D1 mRNA co-localizes with the caprin-1, G3BP1, and c-Myc mRNA-associated RNP complex (Fig. 2B), as well as within polysomal fractions (Fig. 2D). These results indicate that either cyclin D1 or D2 mRNAs might be sequestered by biotinylated tylophorine (Fig. 2B & 2D) or associated with a tylophorine-targeted RNP complex in tylophorine-treated HONE-1 cells (Fig. 2D).

### Tylophorine represses the protein expression of c-Myc and cyclins D1/D2 but does not decrease their mRNA levels through association with caprin-1

We examined the effect of tylophorine on the expression of c-Myc and cyclins D1/D2 based on the above pull-down results. Tylophorine treatment increased the mRNA levels of c-Myc and cyclin D1 in both HONE-1 and MCF7 cells. Cyclin D2 mRNA levels were increased only in HONE-1 cells upon tylophorine treatment since cyclin D2 mRNA level was undetectable in MCF7 cells as reported [30-32], regardless of tylophorine treatment (Fig. 3A). However, c-Myc and cyclin D1 protein levels were diminished in HONE-1, MCF7, or NUGC-3 cells upon tylophorine treatment (Fig. 3B). In addition, and as expected, cyclin D2 protein levels were decreased in tylophorine treated HONE-1 and NUGC-3 but no cyclin D2 were detectable in MCF7 cells (Fig. 3B). Furthermore, we used TAMRA to label the newly synthesized protein after tylophorine treatment and followed by immunoprecipitation and western blot to detect the proteins of interest. We found that the de novo protein syntheses of c-Myc and cyclinD1/D2 were diminished upon tylophorine treatment but not that of G3BP1 protein (Fig. 3C). When HONE-1 cells were depleted with caprin-1 by siRNA, the resultant cells became more resistant to tylophorine compounds with an ~2 fold higher IC_50_ value for tylophorine and its potent derivative DBQ 33b (dibenzoquinoline-33b) (Fig. 3D). Accordingly, the amounts of G3BP1 protein (Fig. 3E) and mRNAs of c-Myc and cyclins D1/D2 pulled down by biotinylated tylophorine were decreased (Fig. 3F).

These results suggest that tylophorine blocked the protein expressions of c-Myc and cyclins D1/D2 through association with caprin-1 thereby downregulating their downstream signaling pathways to exert the anti-cancer activity. The roles of tylophorine-downregulated signaling pathways of c-Myc and cyclins D1/D2 in anti-cancerous tylophorine were investigated as followed.

### Tylophorine hypophosphorylates and activates pRb through repressing protein expression of cyclins D1/D2 and c-Myc

**Figure 4:**
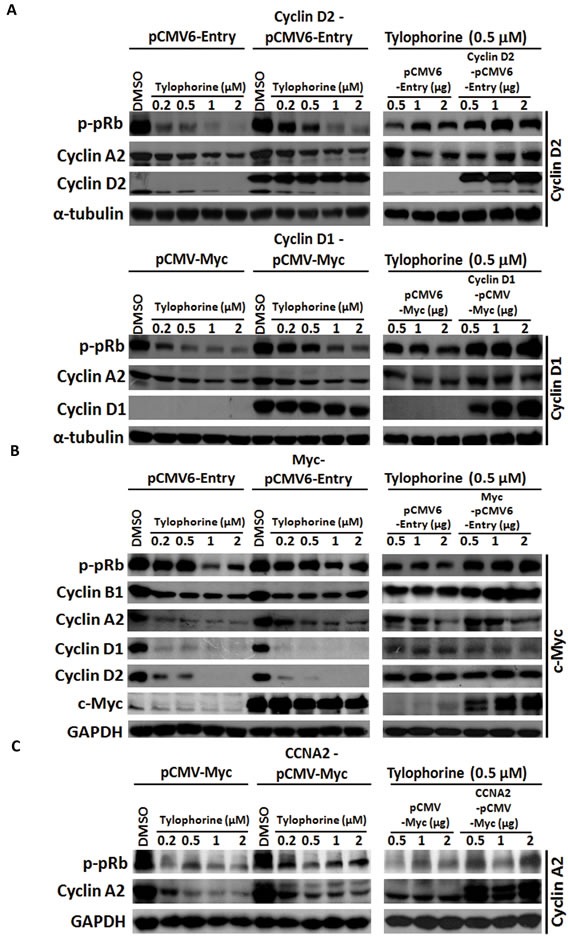
Gain-of-function experiments for tylophorine-induced decreased protein expression of cyclin D1, cyclin D2, and c-Myc A. Ectopically overexpressed cyclin D1 or D2 restored the hyperphosphorylation of pRb and the expression of cyclin A2. B. Ectopically overexpressed c-Myc rescued the biological function of its downstream effectors (e.g., cyclin B1 and p-pRb) but not the protein expression of cyclin D1 and D2. C. Ectopically overexpressed cyclin A2 moderately restored the hyperphosphorylation of pRb. HONE-1 cells were transfected with the indicated expression vectors for 24 h prior to treatment with either vehicle (DMSO) or tylophorine at the indicated concentrations for an additional 24 h. The resulting cell lysates were analyzed by western blotting with the indicated antibodies. The relative expression or phosphorylation levels of each protein were normalized with their respective internal loading control α-tubulin or GAPDH. The results shown are representative of 3 independent experiments.

pRb protein, a common downstream effector of cyclins D1 [8, 9] and D2 [10, 11] and c-Myc [1, 7], was further examined for tylophorine effects on the anticancer activity. pRb was hypophosphorylated to the active form in tylophorine-treated carcinoma cells, whereas its protein expression levels remained unchanged (Fig. 3B); this result was expected because the protein expressions of the pRb's upstream regulators, cyclins D1 [8, 9]/D2 [10, 11] and c-Myc 1, 7], were reduced by tylophorine treatment thereby decreasing the phosphorylation of pRb (Fig. 3B). Cyclin A2 was downregulated by tylophorine, which partially accounts for the G1 arrest induced by tylophorine [20, 25]. The cyclin A2 promoter region contains two E2F regulatory sites. When either cyclin D2 (Fig. 4A-upper panel) or cyclin D1 (Fig. 4A-lower panel) was overexpressed, the downstream hyperphosphorylation of pRb resumed, thereby upregulating E2F-mediated cyclin A2 expression [35] in tylophorine-treated cells (Fig. 4A).

Moreover, ectopic overexpression of c-Myc also restored the hyperphosphorylation of the downstream effector pRb as well as cyclin A2 expression. Strikingly, the expression of cyclins D1/D2 was not restored by ectopic overexpression of c-Myc (Fig. 4B), even though cyclins D1/D2 are downstream target genes of c-Myc signaling [36, 37]. The resulting hyperphosphorylation of pRb can be, at least in part, attributed to increased levels of cyclin B1 [38] (Fig. 4B), a c-Myc target and another upstream effector of pRb, or feedback from upregulated cyclin A2, as ectopic overexpression of cyclin A2 was also able to partially resume the hyperphosphorylation of pRb (Fig. 4C). These results further emphasize that even ectopically expressed c-Myc can upregulate or stabilize the transcription of cyclins D1 and D2, but their mRNA is subject to sequestration by tylophorine in the caprin-1 and c-Myc mRNA-associated RNP complex, thereby blocking their translation. Therefore, ectopically expressed c-Myc resumed the hyperphosphorylation of pRb through a pathway independent of cyclins D1/D2.

In summary, active hypophosphorylated pRb can be inactivated to hyperphosphorylated form upon the overexpression of cyclins D1/D2 or c-Myc in tylophorine-treated carcinoma cells. The hypophosphorylation of pRb plays an important role in facilitating the anti-cancer activity of tylophorine. Accordingly, the downstream pathway targets of c-Myc as well as those of cyclins D1 and D2 (e.g. pRb, cyclin A2 and cyclin B1) were also downregulated and contributed to the anti-cancer activity of tylophorine.

### The effects of tylophorine treatment on cellular events specifically regulated by c-Myc such as the Warburg effect in cancer metabolism and formation of processing bodies

To demonstrate the functional consequences of c-Myc signaling inhibition by tylophorine, cellular events specifically regulated by c-Myc such as the Warburg effect in cancer metabolism, and formation of processing bodies (P-bodies), were examined.

**Figure 5:**
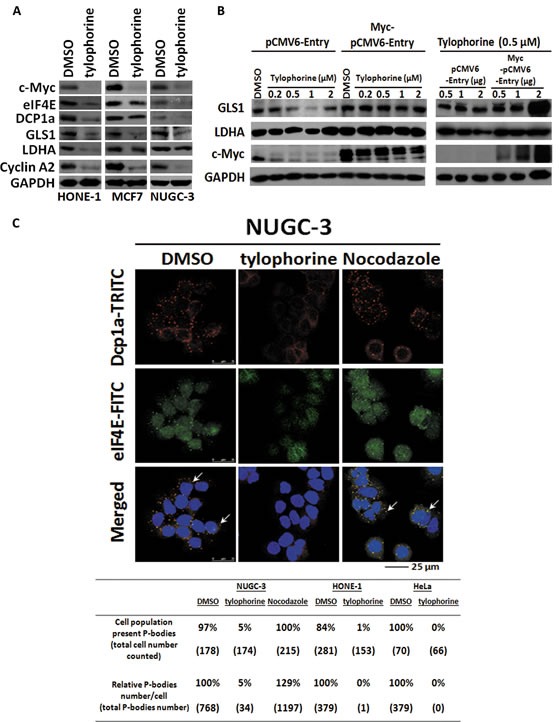
The effects of tylophorine compounds in c-Myc regulated Warburg effect and P-body (processing body) formation A. Tylophorine treatment decreased the protein expressions of GLS1 and LDHA involved in Warburg effect as well as eIF4E and DCP1a in P-body formation. B Ectopically Overexpressed c-Myc restored the protein expressions of GLS1 and LDHA. The relative expression levels of each protein were normalized with their respective internal control GAPDH. C. Tylophorine treatment decreased the formation of P-bodies. The P-bodies were visualized by immunofluorescent staining using anti-Dcp1a-TRITC (in red) and eIF4E-FITC (in green). DAPI (in blue) was used for nuclear counterstaining. The carcinoma cells were treated with DMSO, tylophorine (2 μM), or nocodazole (30 ng/mL) for 24 h prior to western blotting or immunofluorescent staining analyses with the indicated antibodies. The percentage of the cell population exhibiting P-bodies and the relative percentages of the total number of P-bodies in each treatment were counted with Image-J software (National Institutes of Health) and listed. Nocodazole was used an additional compound control that induces formation of P-bodies. The results shown are representative of 3 independent experiments.

Although most tylophorine-treated cells were viable, their proliferation was completely suppressed [20], and no significant apoptosis occurred [25]. Therefore, we examined the effect of tylophorine treatment on GLS1 (Glutaminase type 1) [3] and LDHA (Lactate dehydrogenase) [2], c-Myc-targeted genes that mediate the cancer cell nutrient supply via the Warburg effect, and observed that the protein expression levels of these genes were downregulated in tylophorine-treated carcinoma cells (Fig. 5A and 5B). When c-Myc was overexpressed in tylophorine-treated HONE-1 cells, the protein expression levels of GLS1 and LDHA were restored (Fig. 5B). Thus, tylophorine downregulated the Warburg effect by suppressing c-Myc protein expression levels.

The levels of eIF4E [4, 5] (a c-Myc target gene) which is involved in cytoplasmic mRNP processing, were significantly reduced in carcinoma cells, as were the levels of Dcp1a, another constituent of P-bodies [39-41] (Fig. 5A). We next examined the effects of tylophorine treatment on P-body formation using confocal immunofluorescent microscopy, which indicated that both the numbers of P-bodies and the populations of cells that exhibited P-bodies were dramatically decreased to 0~5% upon tylophorine treatment in HONE-1, NUGC-3, or HeLa cells (Fig. 5C).

### Tylophorine downregulates c-Myc protein expression levels in carcinoma cells *in vitro* and *in vivo*

**Figure 6:**
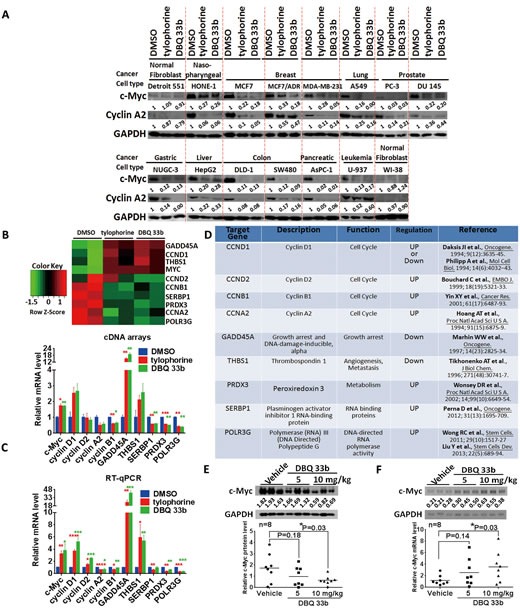
The tylophorine compounds downregulate c-Myc protein levels and affect the expression of c-Myc target genes in carcinoma cells treated with tylophorine compounds *in vitro* or in *vivo* A. Tylophorine and a tylophorine-derived-dibenzoquinoline 33b (DBQ 33b) inhibited the protein expression of c-Myc in 13 carcinoma and 2 fibroblast cell lines. The results shown are representative of 3 independent experiments. B-D. The effects of tylophorine on the expression of c-Myc target genes. The results were obtained from an analysis of gene expression profiling with a cDNA-array (B) and verified by RT-qPCR (C). The genes regulated by the tylophorine compounds through c-Myc are listed by function and their upregulation or downregulation by c-Myc as determined in previous studies as indicated (D). Cells were treated with vehicle (0.1% DMSO), tylophorine (2 μM), or DBQ 33b (0.5 μM) for 24 h prior to lysis for western analysis with the indicated antibodies (A) or prior to mRNA extraction for gene expression profiling using the Illumina-HumanHT-12-v4 Expression-BeadChip (B) and RT-qPCR analysis (C). E & F. The protein (E) and mRNA (F) expression levels of c-Myc were downregulated *in vivo* in A549 xenograft tumors in mice by treatment with DBQ 33b [22]. (E) The protein expression of c-Myc detected by immunoblot analysis (upper panel) was quantitated and shown individually (lower panel). (F) The mRNA expression of c-Myc was analyzed by RT-qPCR; the products were shown in the upper panel, and the individual data were plotted in the lower panel. The relative expression levels of mRNA or protein were normalized with their respective internal loading control GAPDH. The sequences of the gene primer pairs used were listed in Supplemental Table 3. *, p<0.05; **, p<0.005; ***, p<0.0005; ****, p<0.0001.

Because tylophorine-related alkaloids potently inhibit the growth of a wide variety of drug-sensitive and drug-resistant cancer cell lines, we evaluated c-Myc protein expression levels in a panel of carcinoma cell lines (13 carcinoma cell lines from 9 types of tumors and 2 fibroblast cell lines) with tylophorine treatment. We observed the sustained blockade of c-Myc protein expression in each of the individual carcinoma cell lines upon treatment with tylophorine or its potent derivative DBQ 33b [23] but not in the fibroblast cell lines. c-Myc protein expression levels were reduced to 1-33% (Fig. 6A), whereas c-Myc mRNA levels were increased by 1.5-4.5-fold (Figs. 3A, 6B, & 6C). We also performed gene expression profiling and analysis of the c-Myc downstream pathways that were affected by tylophorine or DBQ 33b (Fig. 6B), as well as validation by RT-qPCR (Fig. 6C). Both compounds increased the expression of c-Myc mRNA by ~2-4-fold; the mRNA levels of genes that were upregulated by c-Myc were decreased (e.g., cyclin A2, cyclin B1, SERBP1, PRDX3, and POLR3G), and the mRNA levels of genes that were downregulated by c-Myc increased (e.g., GADD45A, and THBS1) (Fig. 6B and 6C). These results are consistent with the above observation that increased c-Myc mRNA levels and decreased c-Myc protein levels occurred in tylophorine-treated cells, which led to negative regulation of c-Myc effector pathways. The genes, whose expression was upregulated or downregulated by tylophorine and DBQ 33b, associated with the function of c-Myc in carcinogenesis are shown in Fig. 6D.

In a mouse tumor model using human lung A549 carcinoma xenografts, DBQ 33b significantly reduced the tumor volume in a dose-dependent manner [23]. Finally, we examined the c-Myc protein and its mRNA expression in these xenograft tumors [23]. Our results further confirmed reductions in c-Myc protein levels with concomitant increases in its mRNA levels in a dose-dependent manner in the DBQ 33b-treated tumors compared to the vehicle-treated tumors (Fig. 6E and 6F). These results correlated with the *in vivo* anti-tumor activity [23] and *in vitro* anti-cancer results for DBQ 33b (Fig. 6A, 6B, & 6C).

## DISCUSSION

**Figure 7:**
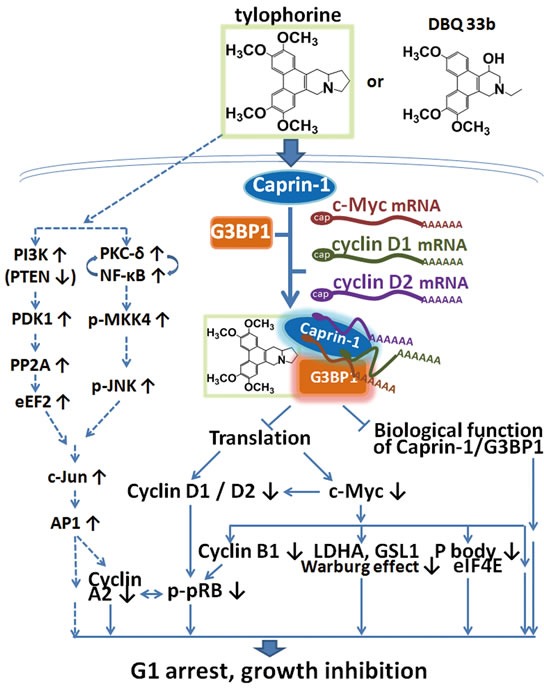
A summary scheme for tylophorine-targeted anti-cancer pathways The solid lines indicate the direct targeting of tylophorine compounds to caprin-1, c-Myc, and the related pathways. The dashed lines indicate the previously published c-Jun-mediated anti-cancer mechanism of tylophorine[25] including the results shown in Supplemental Figure 2 for decreased PTEN. The elevation of c-Jun by tylophorine compounds is not affected by ectopically overexpressed c-Myc as shown in Supplemental Figure 3 indicating that the tylophorine compounds target the caprin-1 and c-Myc mRNA-containing RNP complexes in parallel with their effects on c-Jun accumulation to elicit anti-cancer activity.

In this study, it was found that tylophorine compounds exert their anti-cancer activities via targeting the caprin-1, G3BP1, c-Myc mRNA, and cyclin D2 or D1 mRNAs containing RNP complex, inhibiting the functions of the RNP components and blocking the protein translation of the corresponding mRNA transcripts (e.g., c-Myc, cyclin D2, and cyclin D1, as well as their downstream pathway components such as pRb) (Fig. 7). Because abnormal c-Myc signaling in cancer cells has been associated with oncogenicity, the downregulation of c-Myc by tylophorine compounds might play a significant role in the anti-cancer activity of these compounds.

The therapeutic targeting of RNP complexes represents an under-explored but emerging strategy for drug discovery [42]. RNPs are ribonucleoproteins in which RNA and protein molecules, such as the ribosome, the enzyme telomerase, hnRNP, small nuclear RNPs (snRNPs), and viral RNP complexes, interact. RNP complexes exhibit multiple roles, dynamic conformations, and chemical instability. Therefore, targeting RNPs for the discovery or development of therapeutic agents remains a challenge. However, several antiviral agents have been reported to target viral RNP complexes with high potency and exhibit significant potential for development into therapeutic drugs [43-45]. To the best of our knowledge, no anti-cancer agent targeting an RNP complex has been reported. We are the first to report that targeting an RNP complex (i.e., targeting the caprin-1 and c-Myc mRNA-containing RNP complex by tylophorine compounds) in carcinoma cells can efficiently elicit anti-cancer activity.

Significantly, pull-down experiments using biotinylated tylophorine revealed the association of caprin-1, G3BP1, c-Myc mRNA, and cyclins D1/D2 mRNA in untreated carcinoma cell lysates (Fig. 1D and 2B) in which the mRNAs for c-Myc and cyclin D1 and D2 were not upregulated, as was observed in the tylophorine-treated carcinoma cells (Fig. 2D-a). However, we did not observe the polysomal colocalization of G3BP1, c-Myc mRNA, and cyclins D1/D2 mRNA with caprin-1 in the DMSO-treated carcinoma cells by sedimentation fractionation (Fig. 2D-b). Thus, we conclude that the binding of tylophorine to caprin-1 enhanced the recruitment of G3BP1, c-Myc mRNA, and cyclins D1/D2 mRNA to the RNP complex. This hypothesis was also confirmed by the association of caprin-1 with G3BP1, c-Myc mRNA, and cyclins D1/D2 in polysomal fractions only in tylophorine- but not vehicle-treated carcinoma cells (Fig. 2D), whereas the c-Myc mRNA and cyclins D1/D2 mRNA levels were elevated in tylophorine-treated carcinoma cells (Fig. 2D-a and 3A).

The increased mRNA levels of c-Myc and cyclins D1 and D2 induced upon tylophorine treatment of the carcinoma cells might at least partially reflect an impairment of mRNA degradation due to tylophorine treatment, consistent with the dramatic decrease observed in the number of P-bodies, which suggested a blockade of cellular mRNA degradation processes. Moreover, cytoplasmic mRNAs cycle between polysomes, P-bodies and stress granules and translational repress can occur in these compartments through different mechanisms [46]. In addition to tylophorine treatment dramatically decreased the formation of P-bodies (Fig. 5C), stress granules were nearly not found either in DMSO or tylophorine treated carcinoma cells (Supplemental Figure 1). Using TAMRA to label the newly synthesized protein by tylophorine treatment, we clearly demonstrated that tylophorine repressed the protein expression of c-Myc and cyclins D1/D2 (Fig. 3C). These results further firm that tylophorine sequestered the RNP containing caprin-1 and c-Myc mRNA in the polysomal fractions (Fig. 2D) and blocked the corresponding mRNAs' translation. However, the detailed underlying mechanisms must be carefully addressed and further investigated. Our results suggest that caprin-1 is highly associated with the transport of c-Myc, cyclin D1, and cyclin D2 mRNAs for translation. Thus, the abrogation of this specific biological function of caprin-1 results in the induction of cell cycle arrest at the G1 phase [47].

The expression of c-Myc is elevated in numerous tumor types as the result of multiple mechanisms. c-Myc accumulates at the promoter regions of active genes in carcinoma cells, leading to their abnormal transcriptional amplification and oncogenicity [48]. Therefore, the upregulation of c-Myc is generally associated with tumor aggressiveness and poor clinical outcome. Thus, it is conceivable that the inhibition of c-Myc significantly contributes to the broad-spectrum anti-cancer activity of tylophorine compounds against drug-resistant carcinoma cells. Moreover, the induction of cell cycle arrest at G1 [47] by knockdown of caprin-1 suggests that, in addition to the caprin-1 and c-Myc mRNA-associated RNP complex, caprin-1 itself may have favorable potential as a novel anti-cancer drug target.

Ultimately, the blockade of c-Myc mRNA transport by the tylophorine-mediated sequester of the caprin-1, G3BP1, c-Myc mRNA, and cyclin D2 mRNA-containing RNP complex may contribute significantly to the anti-cancer effects elicited by tylophorine in conjunction with the accumulation of c-Jun [25] (Fig. 7). Tylophorine enhances the c-Jun downregulation of the cyclin A2 promoter activity and results in carcinoma cells dominantly arrested at G1 phase for anti-cancer activity [25]. The ectopically overexpressed c-Myc did not affect the accumulation of c-Jun induced by tylophorine treatment but did increase the expression of cyclin A2 via hyperphosphorylated pRb (Supplemental Figure 2 and Fig. 4B). Thus, we conclude that the elevation of c-Jun levels by tylophorine [23, 25] functions in concert with the targeting of the caprin-1 and c-Myc mRNA-containing RNP complex to produce the anti-cancer activity of tylophorine compounds (Fig. 7).

## MATERIALS AND METHODS

### Tylophorine compounds and biotinylated tylophorine (BT)

Tylophorine and DBQ 33b were prepared as described with a purity greater than 95% as determined by reverse pahse-HPLC [23]. The scheme for synthesis of biotinylated tylophorine, with purity of 95.87% analyzed by reverse pahse-HPLC, was shown in Fig. 1A (see NMR data below and Supplemental Figure 4).

Compound 1 was prepared as previously described [49] and reacted overnight with a mixture of NaH and tert-butyl (2-bromoethyl) carbamate in DMF to produce compound 2. We reduced compound 2 with LiAlH_4_ and AlCl_3_ (at a 3:1 ratio) in THF at room temperature for 4 h to produce compound 3. The *N*-Boc-protected amine was deprotected with H_2_SO_4_ in CH_2_Cl_2_ at room temperature for 22 h to produce compound 4. Finally, Biotin-X-SSE (Invitrogen) was coupled to compound 4 using Et_3_N in DMF at room temperature for 22 h to produce compound 5, the biotinylated tylophorine, a tylophorine-based dibenzoquinoline derivative (Fig. 1A). Compound 5: Yellow crystal; ^1^H-NMR (300 MHz, CD_3_OD): 1.30-1.70 (12H, m), 2.13 (2H, t, *J*=7.2 Hz), 2.24 (2H, m), 2.65 (1H, d, *J*=12.9 Hz), 2.85 (2H, dd, *J*=12.9 Hz, *J*=4.8 Hz), 2.99 (2H, d, *J*=5.6 Hz), 3.11 (4H, t, *J*=7.2 Hz), 3.41 (2H,br d, *J*=16.8 Hz), 3.69 (2H, t, *J*=5.6 Hz), 4.02 (3H, s), 4.04 (3H, s), 4.08 (6H, s), 4.22 (1H, dd, *J*=8.0 Hz, *J*=4.5 Hz), 4.41 (1H, dd, *J*=8.0 Hz, *J*=4.5 Hz), 4.50 (2H, s), 7.20 (1H, s), 7.34 (1H, s), 7.93 (1H, s), 7.94 (1H, s). ^13^C-NMR (500 MHz, CDCl_3_): 22.3, 24.5, 25.3, 26.1, 27.6, 27.8, 28.3, 29.6, 34.0, 35.3, 36.0, 38.5, 40.6, 48.6, 51.1, 53.9, 55.6, 56.0, 56.2, 59.7, 61.9, 102.7, 103.2, 103.4, 103.6, 117.7, 122.7, 123.8, 124.0, 124.3, 149.2, 149.3, 149.6, 163.7, 173.6, 175.4. ESI-MS *m/z* 758 (M + Na)^+^; HRMS calcd for C_39_H_53_O_7_N_5_NaS, 758.35579 (M^+^); found, 758.35592.

### Cell lines and culture

Two human fibroblast cell lines (Detroit 551 [ATCC CCL-110]; WI-38 [ATCC CCL-75]), the human embryonic kidney cell line (HEK-293[ATCC CRL-1573]), and 14 human carcinoma cell lines (breast, MCF7 [ATCC HTB-22], MCF7/ADR [ATCC], MDA-MB-231 [BCRC 60425]; cervix, HeLa [BCRC 60005]; colon, DLD-1 [ATCC CCL-221], SW480 [ATCC CCL-228]; gastric, NUGC-3 [the Japanese Cancer Research Resources Bank]; leukemia, U-937 [ATCC CRL-1593.>2]; liver, HepG2 [ATCC HB-8065]; lung, A549 [ATCC CCL-185](obtained from American Type Culture Collection and cultured for passages in fewer than 6 months from initial purchase); nasopharyngeal, HONE-1 [a gift from Dr. Ching-Hwa Tsai at National Taiwan University, Taiwan, ROC] (short tandem repeat (STR) profile was performed by Bioresource Collection and Research center (BCRC), Taiwan; The DNA profile was unique and no match was found in any known STR database, see data in Supplemental Table 4) ; pancreatic, AsPC-1[BCRC 60494]; and prostate, DU 145 [ATCC HTB-81], PC-3 [BCRC60122]) were used in this study. HEK-293, HeLa, HONE-1, MCF7, MCF7/ADR, HepG2, PC-3, NUGC-3, AsPC-1, Detroit 551, and WI-38 cells were maintained in Dulbecco's Modified Eagle's Medium (DMEM, Hyclone Laboratory Inc.) supplemented with 10% fetal bovine serum (FBS, Hyclone Laboratory Inc.). DU 145 and U-937 cells were maintained in DMEM-FBS medium supplemented with 1 mM sodium pyruvate (GIBCO-Life Technologies). DLD-1, A549, and MDA-MB-231 cells were cultured in RPMI 1640 medium (GIBCO-Life Technologies) supplemented with 10% FBS, and SW480 cells were maintained in RPMI 1640-FBS medium supplemented with 1 mM sodium pyruvate.

### Cell Growth Inhibitory Assays

HONE-1, caprin-1 silencing HONE-1 cells (3000 cells/well), MCF-7 (6500 cells/well ) and NUGC-3 (4500 cells/well) were seeded into 96-well plates and subjected to measurement of growth inhibition by tylophorine, biotinlylated tylophorine, or DBQ 33b and the IC_50_ values were determined as previously described [18].

### Pull-down assay and in-gel digestion

HONE-1 cell lysates were incubated with biotinylated tylophorine at 4°C for 4 h. The biotinylated tylophorine-associated complexes were then pulled down using streptavidin Dynabeads M280 (Invitrogen) at 4°C for 1.5 h. The biotinylated tylophorine-bound proteins were eluted with Laemmli buffer and analyzed by electrophoresis on a 4%–20% gradient SDS-PAGE gel (W/H: 16 cm × 15 cm) and visualized by silver staining (Amersham Biosciences). The protein bands of interest were excised, subjected to in-gel digestion (see Supplemental Materials and Methods), and analyzed by LC/MS/MS using a Thermo Scientific LTQ XL mass spectrometer (Thermo Scientific). In addition, western blot was used to identify the protein components from the pull-down. The biotinylated tylophorine-bound RNAs were eluted with TRizol Reagent (Invitrogen) and RT-PCR was further used for analyzing the associated components.

### Reverse transcription (RT) and quantitative polymerase chain reaction (qPCR)

The previously described procedure was used [21]. The mRNA expression levels in Fig. 2B, 2D, and 3A were determined by semi-RT-qPCR, analyzed using the Gel-Pro Analyzer program, and normalized using the housekeeping gene GAPDH when necessary. In Fig. 6, the changes in mRNA expression levels were determined using the ΔΔCT method with GAPDH housekeeping genes. The primer pairs used in the PCR reactions are listed in Supplemental Table 3.

### Western analyses and co-immunoprecipitation

We performed western blotting as previously described [18, 20] with antibody listed in Supplemental Table 5. See Supplemental Materials and Methods for co-immunoprecipitation using anti-caprin-1 antibody (ProteinTech Group).

### Purification of recombinant human caprin-1 expressed in HEK-293 cells

CAPRIN1-pCMV6-entry was obtained from Origene, and transfected in HEK-293 cells. Expressed caprin-1 was purified using anti-FLAG M2 affinity gel (Sigma-Aldrich). After washes with TBS buffer, the human caprin-1 protein was eluted with 3X Flag peptide (N-Met-Asp-Tyr-Lys-Asp-His-Asp-Gly-Asp-Tyr-Lys-Asp-His-Asp-Ile-Asp-Tyr-Lys-Asp-Asp-Asp-Asp-Lys-C, Sigma-Aldrich).

### Polysome profile analysis

HONE-1 cells were treated with 50 μg/ml cycloheximide at 37°C for 30 min before harvest. Total cell lysates were clarified by centrifugation at 4°C for 10 min at 13,000 rpm. The supernatants were loaded on the top of a 10-50% sucrose gradient and ultracentrifuged in a SW41Ti Beckman rotor at 4°C for 4 h at 36,000 rpm. The fractions were collected from the bottom of the tubes, and the absorbance was measured at a wavelength of 254 nm using an AKTA purifier (GE Healthcare, Amersham Biosciences). RNA and protein were extracted using TRIzol reagent (Invitrogen). Protein expression and mRNA expression were further analyzed by western immunoblot analysis and semi-RT-qPCR, respectively.

### TAMRA Labeling and Detection of De Novo Synthesized Proteins of Interest

HONE-1 cells were washed with PBS and cultured with methionine-free DMEM (GIBCO-Life Technologies) at 37°C for 1 h prior to the addition of 2 μM tylophorine and 25 μM Click-iT® HPG (L-homopropargylglycine, Invitrogen) and incubation at 37°C for 24 h. The cells were then harvested and subjected to the Click Reaction with 20 μM TAMRA (Tetramethylrhodamine, Invitrogen) for labeling the de novo synthesized proteins in Click-iT® Protein Reaction Buffer according to the manufacturers' protocol (Invitrogen). The resultant cell lysates were immunoprecipitated with anti-caprin-1 (ProteinTech Group), anti-c-Myc (Cell Signaling Technology), anti-cyclin D1, anti-cyclin D2 (Santa Cruz Biotechnology), anti-TAMRA (Thermo Scientific) or GAR (Perkin-Elmer) respectively overnight at 4°C with constant agitation prior to incubation with protein G agarose (Millipore) at 4°C for another 2 h. After washes, the specific immunoprecipitated protein was eluted and analyzed by western immunoblot analysis with the antibody indicated.

### CAPRIN1 Gene Silencing

HONE-1 cells were transfected with CAPRIN1 shRNA plasmid (TL312677, origene), or scrambled pGFP-C-shLenti shRNA (TR30021, origene) using FuGene 6^TM^ (Roche). At 72 h post transfection, the cells were cultured in the presence of 1.5 mg/mL puromycin for selection. In addition, pseudotyped lentivirus containing CAPRIN1 shRNA (clone ID: TRCN0000115975 and TRCN0000115976) or negative control shRNA (shLacZ, clone ID: TRCN72224)(Academia Sinica, Taiwan) were transducted into HONE-1 cells with MOI of 3 in culture medium containing 8 μg/ml polybrene (Millipore). At 24 h post transduction, the cells were cultured in the presence of 1.5 mg/mL puromycin for selection.

### Transfection

FuGene 6^TM^ (Roche) was used for transient transfection of the constructs into HONE-1 cells according to the manufacturers' protocols. At 24 h after transfection, the HONE-1 cells were treated with either the vehicle control (0.1% DMSO) or 0.2 to 2 μM tylophorine for an additional 24 h. The cells were then harvested in lysis buffer as described above prior to immunoblot analyses.

### Plasmid constructs

The coding regions of human cyclin D1, cyclin D2, and c-Myc were amplified from HONE-1 cDNA using the following primer pairs: 5′-CGGAATTCGGATGGAACACCAGCTCCTGTG-3′ & 5′-CCGCTCGAGTCAGATGTCCACGTCCCG-3′ for cyclin D1; 5′-AATGCAGCGATCGCCAT GGAGCTGCTGTGCCACGAGG-3′& 5′-GTGACGACGCGTCAGGTCGATATCCCGCACGTC TGTA-3′ for cyclin D2; and 5′-AATGCAGCGATCGCCCTGGATTTTTTTCGGGTA GTGGAAAACCAGCAGCCTCC-3′& 5′-GTGACGAC GCGTCGCACAAGAGTTCCGTAGCT GTTCAAGT-3′ for c-Myc. The coding regions were next subcloned into the EcoRI/XhoI site of pCMV-Myc and the AsiSI/MluI site of pCMV6-Entry to generate the CCND1-pCMV-Myc, CCND2-pCMV6-Entry, and MYC-pCMV6-Entry expression vectors. The human CCNA2-pCMV-myc expression vector [20] was amplified from the MGC 132447 clone (Open Biosystems) with primer pairs of 5′-CGAATTCCGATGTTGGGCAACTCTGCG-3′ and 5′-GCCTCGAGTTACAGATTTAGTGTCTCTGGTG G-3′, then subcloned into EcoRI/XhoI sites of pCMV-myc (Clontech).

### Immunofluorescent assay (IFA) and confocal laser scanning microscopy

IFA were performed as described previously [21]. We used anti-DCP1a, anti-eIF4E, anti-G3BP1 (Santa Cruz Biotechnology) and anti-PABP (Abcam) antibodies to detect P-bodies and stress granules. Fluorescence imaging was performed using a Leica TSC SP5 laser-scanning confocal microscope.

### Protein extraction from xenografted murine tumors

The xenografted murine tumor tissues [23] were flash-frozen in liquid nitrogen, washed with ice-cold PBS, and homogenized using a TissueLyser II (Qiagen) according to the manufacturer's protocols. Total protein was extracted in lysis buffer as detailed above and further analyzed by western blotting. Total RNA was extracted using TRIzol Reagent (Invitrogen), reverse transcribed using Superscript III (Invitrogen), and analyzed by real-time RT-qPCR as described above.

### Gene expression profiling using cDNA arrays

HONE-1 cells were treated with the vehicle control (0.1% DMSO), 0.5 μM DBQ 33b, or 2 μM tylophorine. After treatment with the compounds for 24 h, total RNA was extracted using TRIzol reagent (Invitrogen), and expression profiling of the coding genes performed using the Illumina Human HT-12 v4 Expression BeadChip. Furthermore, the gene expression data from the Illumina arrays were validated by real-time RT-qPCR.

## SUPPLEMENTARY MATERIALS AND METHODS, TABLES AND FIGURES


