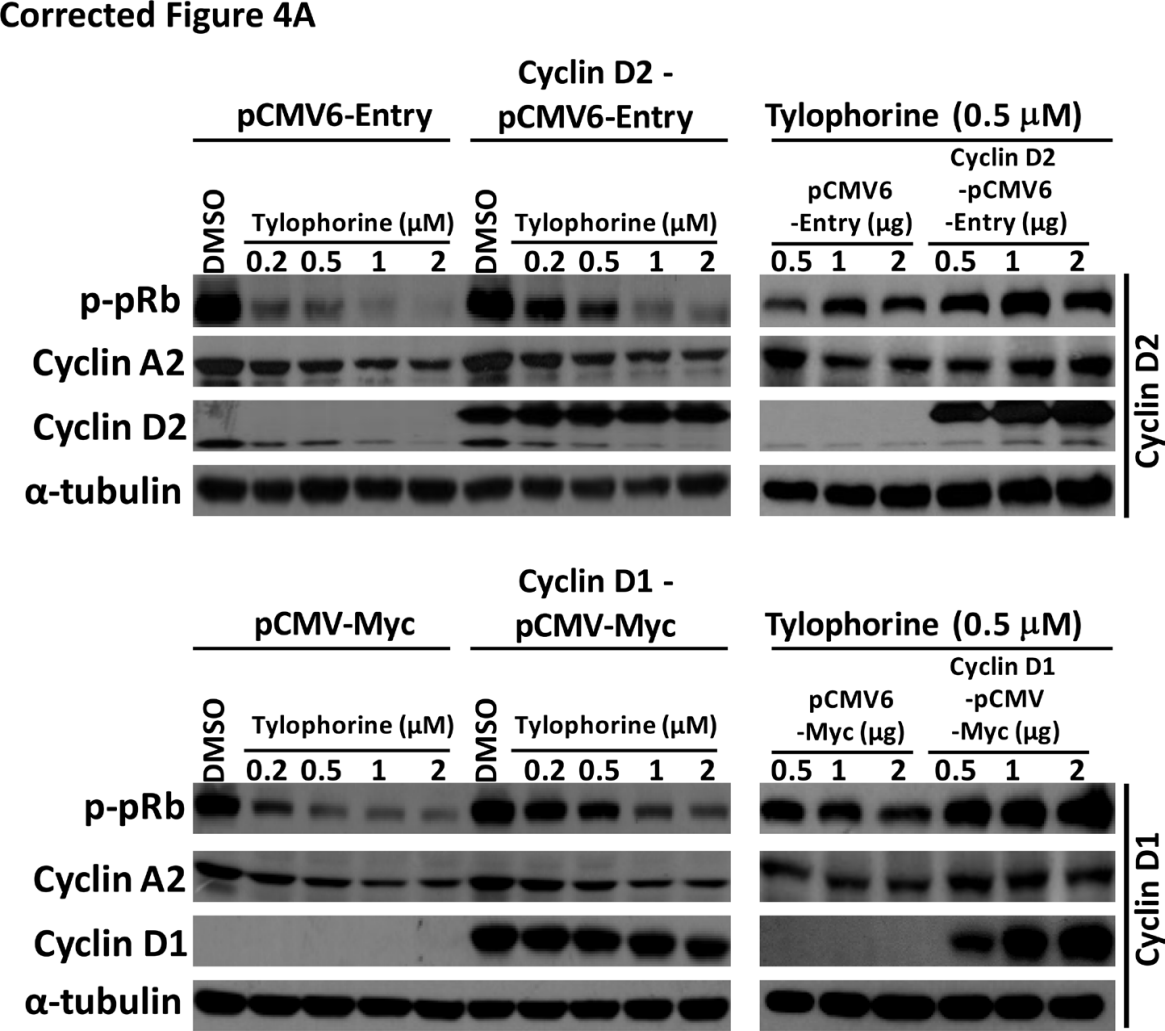# Correction: Targeting a ribonucleoprotein complex containing the caprin-1 protein and the c-Myc mRNA suppresses tumor growth in mice: an identification of a novel oncotarget

**DOI:** 10.18632/oncotarget.27074

**Published:** 2019-08-06

**Authors:** Ya-Qi Qiu, Cheng-Wei Yang, Yue-Zhi Lee, Ruey-Bing Yang, Chih-Hao Lee, Hsing-Yu Hsu, Chien-Chung Chang, Shiow-Ju Lee

**Affiliations:** ^1^ Institute of Biotechnology and Pharmaceutical Research, National Health Research Institutes, Miaoli, Taiwan; ^2^ Graduate Program of Biotechnology in Medicine, Institute of Molecular & Cellular Biology, National Tsing Hua University, Hsinchu, Taiwan; ^3^ Institute of Biomedical Sciences, Academia Sinica, Taipei, Taiwan; ^4^ Department of Genetics and Complex Diseases, Division of Biological Sciences, Harvard School of Public Health, Boston, Massachusetts, USA

**This article has been corrected:** During manuscript editing according to the reviewers' comments, the tubulin control for cyclin D1 in Figure 4A right panel was accidentally misplaced. The corrected Figure 4A is shown below. The authors declare that these corrections do not change the results or conclusions of this paper.

Original article: Oncotarget. 2015; 6:2148–2163. 2148-2163 . https://doi.org/10.18632/oncotarget.13236

**Figure:**