# Supplementary material for: Targeting a ribonucleoprotein complex containing the caprin-1 protein and the c-Myc mRNA suppresses tumor growth in mice: an identification of a novel oncotarget
Source: Oncotarget. 2014 Dec 10;6(4):2148–63. doi: 10.18632/oncotarget.3236 (PMC4385842; doi:10.18632/oncotarget.3236)

Targeting a ribonucleoprotein complex containing the caprin-1 protein and the c-Myc mRNA suppresses tumor growth in mice: an identification of a novel oncotarget

Supplementary Information

Table of Contents

1. **Supplemental Materials and Methods (p.2)**
   1) In-gel digestion of protein samples
   2) Co-immunoprecipitation.
2. **Supplemental Table 1 (p. 2)**
   IC_{50} values for Growth inhibition of biotinylated tylophorine (Compound **5**) against HONE-1, MCF7, and NUGC-3 carcinoma cells.
3. **Supplemental Table 2 (p. 3)**
   Summary of the identified proteins that interacted with the biotinylated tylophorine via the pull-down experiments by MOALDI-TOF-MS and their scores analyzed by Mascot.
4. **Supplemental Table 3 (p. 4)**
   Gene primer pairs used for PCR.
5. **Supplemental Table 4 (p. 5)**
   STR profile of HONE-1 cells.
6. **Supplemental Table 5 (p. 6)**
   Antibodies used for western blots.
7. **Supplemental Figure 1 (p.7)**
   Stress granules were not induced in carcinoma cells after tylophorine treatment.
8. **Supplemental Figure 2 (p. 10)**
   Tylophorine treatment significantly decreased the PTEN protein levels in HONE-1, NUGC-3, and MCF7 carcinoma cells.
9. **Supplemental Figure 3 (p. 10)**
   Ectopically overexpressed c-Myc did not affect the c-Jun accumulation by tylophorine.
10. **Supplemental Figure 4 (p. 11)**
   ^{1}H-NMR and ^{13}C-NMR data of BT.

1. **Supplemental Materials and Methods**

In-gel digestion of protein samples. The gel pieces were washed with ddH_2O and destained in 30 mM potassium ferricyanide (Sigma-Aldrich) and 100 mM sodium thiosulfate (Sigma-Aldrich) for 20 min until they became colorless. Disulfide bonds were reduced by treatment with 10 mM dithiothreitol (DTT, Amersham Biosciences) in 100 mM ammonium bicarbonate (JT Baker) for 1 h at 56°C. Iodoacetamide (Sigma-Aldrich) was then used to alkylate the cysteine residues. The samples were diluted with 50 mM ammonium bicarbonate, dehydrated with acetonitrile (ACN, Echo Chemical), and subjected to 0.001% trypsin (Biological Industries) digestion overnight at 37°C. The next day, the peptide samples were acidified with 5% formic acid (Acros Organics), and analyzed by LC/MS/MS using a Thermo Scientific LTQ XL mass spectrometer (Thermo Scientific).

Co-immunoprecipitation. HONE-1 cell lysates (1500 µg) were incubated with 4 µg of anti-caprin-1 antibody (ProteinTech Group) or GAR (Perkin-Elmer) in PBS containing 10 µM biotinylated tylophorine at 4°C for 4 h with constant agitation. The lysates were then incubated with washed protein G agarose (Millipore) at 4°C for 1.5 h. The beads were washed 5 times with PBS. Caprin-1-bound proteins were then eluted with Laemmli buffer, analyzed by 10% SDS-PAGE, and subjected to western immunoblot analysis.

2. **Supplemental Table 1.** IC_{50} values for Growth inhibition of biotinylated tylophorine (Compound **5**) against HONE-1, MCF7, and NUGC-3 carcinoma cells.

Compound	IC_{50} (µM)		
	HONE-1	MCF7	NUGC-3
Biotinylated tylophorine	30.3 ± 8.5	13.0 ± 4.4	18.7 ± 5.8

3. **Supplemental Table 2.** Summary of the identified proteins that interacted with the biotinylated tylophorine via the pull-down experiments by MOALDI-TOF-MS and their scores analyzed by Mascot.

**Band no.**	**identified protein**	**Masco score**
**1**	α-actinin-4	502
	nucleolin	421
	caprin-1 isoform 1	371
	splicing factor, proline- and glutamine-rich	365
	nucleolar RNA helicase 2	132
	LIM domain and actin-binding protein 1 isoform b	117
	heterogeneous nuclear ribonucleoprotein U isoform a	97
**2**	polyadenylate-binding protein 1	212
	heterogeneous nuclear ribonucleoprotein M isoform a	201
	ATP-dependent RNA helicase DDX3X isoform 1	174
	stress-70 protein, mitochondrial precursor	132
	TATA-binding protein-associated factor 2N isoform 1	92
	heterogeneous nuclear ribonucleoprotein Q isoform 1	83
**3**	heterogeneous nuclear ribonucleoprotein K	267
	ras GTPase-activating protein-binding protein 1	182
	prelamin-A/C isoform 1 precursor	154
**4**	40S ribosomal protein S3a	322
	heterogeneous nuclear ribonucleoprotein A1 isoform b	304
	40S ribosomal protein S3	193
	heterogeneous nuclear ribonucleoproteins A2/B1 isoform B1	115
	40S ribosomal protein S6	63
**5**	40S ribosomal protein S18	202
	60S ribosomal protein L28 isoform 2	195
	40S ribosomal protein S15	191
	40S ribosomal protein S17	144
**6**	40S ribosomal protein S14	297
	40S ribosomal protein S18	163
	40S ribosomal protein S25	139
	60S ribosomal protein L35	110
	60S acidic ribosomal protein P1 isoform 1	108
	60S ribosomal protein L31 isoform 1	104
	60S ribosomal protein L22 proprotein	102

4. **Supplemental Table 3.** Gene primer pairs used for PCR.

Gene Name	Direction	Sequence
**18S**	Forward	GTGGAGCGATTTGTCTGGTT
	Reverse	CGCTGAGCCAGTCAGTGTAG
**28S**	Forward	TGGGTTTTAAGCAGGAGGTG
	Reverse	AACCTGTCTCACGACGGTCT
**Cyclin A2**	Forward	AGCAGCAGAGGCCGAAGAC
	Reverse	ATTAAAAGCCAGGGCATCTTCA
**Cyclin B1**	Forward	GGCTTTCTCTGATGTAATTCTTGC
	Reverse	GTATTTTGGTCTGACTGCTTGC
**Cyclin D1**	Forward	GGTCTGCGCGTGTTTGC
	Reverse	CCCTGACGGCCGAGAAG
**Cyclin D2**	Forward	GATGATCGCAACTGGAAGTG
	Reverse	AGAGACCAGATTATGGACGC
**c-Myc**	Forward	CCAGAGGAGGAACGAGCTAA
	Reverse	AGCCAAGGTTGTGAGGTTGC
**GADD45A**	Forward	AACGGTGATGGCATCTGAAT
	Reverse	CCCTTGGCATCAGTTTCTGT
**POLR3G**	Forward	TTCTCTGCCATCACCCTTTC
	Reverse	TATTCCCAGCCATCAGAACC
**PRDX3**	Forward	CCAGTTCCTCATGCCATGC
	Reverse	TTGACAACGGCTGTACCCTTAA
**SERBP1**	Forward	CTATTCGAGGTCGTGGTGGT
	Reverse	GCCACGAGAATCAAATCCAT
**THBS1**	Forward	CGGTCCAGACACGGACCTGC
	Reverse	GGCTTTGGTCTCCCGCGCTT
**GAPDH**	(RT-PCR)	
	Forward	TACTAGCGGTTTTACGGGCG
	Reverse	TCGAACAGGAGGAGCAGAGAGCGA
	(RT-qPCR)	
	Forward	CGCTCTCTGCTCCTCCTGTT
	Reverse	CCATGGTGTCTGAGCGATGT

5. **Supplemental Table 4.** STR profile of HONE-1 cells.

**Locus**	**HONE-1**
**D8S1179**	12
**D21S11**	27,30
**D7S820**	10,12
**CSF1PO**	10,11
**D3S1358**	15,18
**TH01**	6,7,9
**D13S317**	10,12
**D16S539**	9,10,11
**D2S1338**	17,23
**D19S433**	13
**vWA**	14,16
**TPOX**	8,12
**D18S51**	13,16
**Amelogenin**	X
**D5S818**	11,12
**FGA**	18,21

6. **Supplemental Table 5.** Antibodies used for western blots.

Manufactory	Antibody
**Abcam**	a-actinin-4
	CD82
	DCP1a
	glutaminase (GLS1)
	hnRNP M3-M4
	nucleolin
	PABP
	TAF15
	a-tubulin
**Biosource**	PTEN
**Cell Signaling Technology**	c-Jun
	cyclin B1
	cyclin D1
	cyclin D2
	GAPDH
	LDHA
	p-pRB (S780)
	S3
	S6
	p-S6 (S235/236)
**ProteinTech Group**	caprin-1
**Santa Cruz Biotechnology**	cyclin A2
	c-Myc
	G3BP1
	eIF4E
	hnRNP Q
**Sigma-Aldrich**	PSF

7. **Supplemental Figure 1. Stress granules were not induced in carcinoma cells after tylophorine treatment.** NUGC-3 (**A**), HONE-1 (**B**) and HeLa (**C**) cells were treated with DMSO, 500 μM arsenite, or 2 μM tylophorine for 1.5 h and 24 h. Stress granules assembly were detected by staining with anti-G3BP1-TRITC and PABP-FITC and captured with Leica TSC SP5 laser-scanning confocal microscope. The merged image includes DAPI-stained nuclei. DAPI was used for nuclear counterstaining. The total cell number and stress granules were counted with Image-J software (National Institutes of Health) (D). The results shown are representative of 3 independent experiments.

A

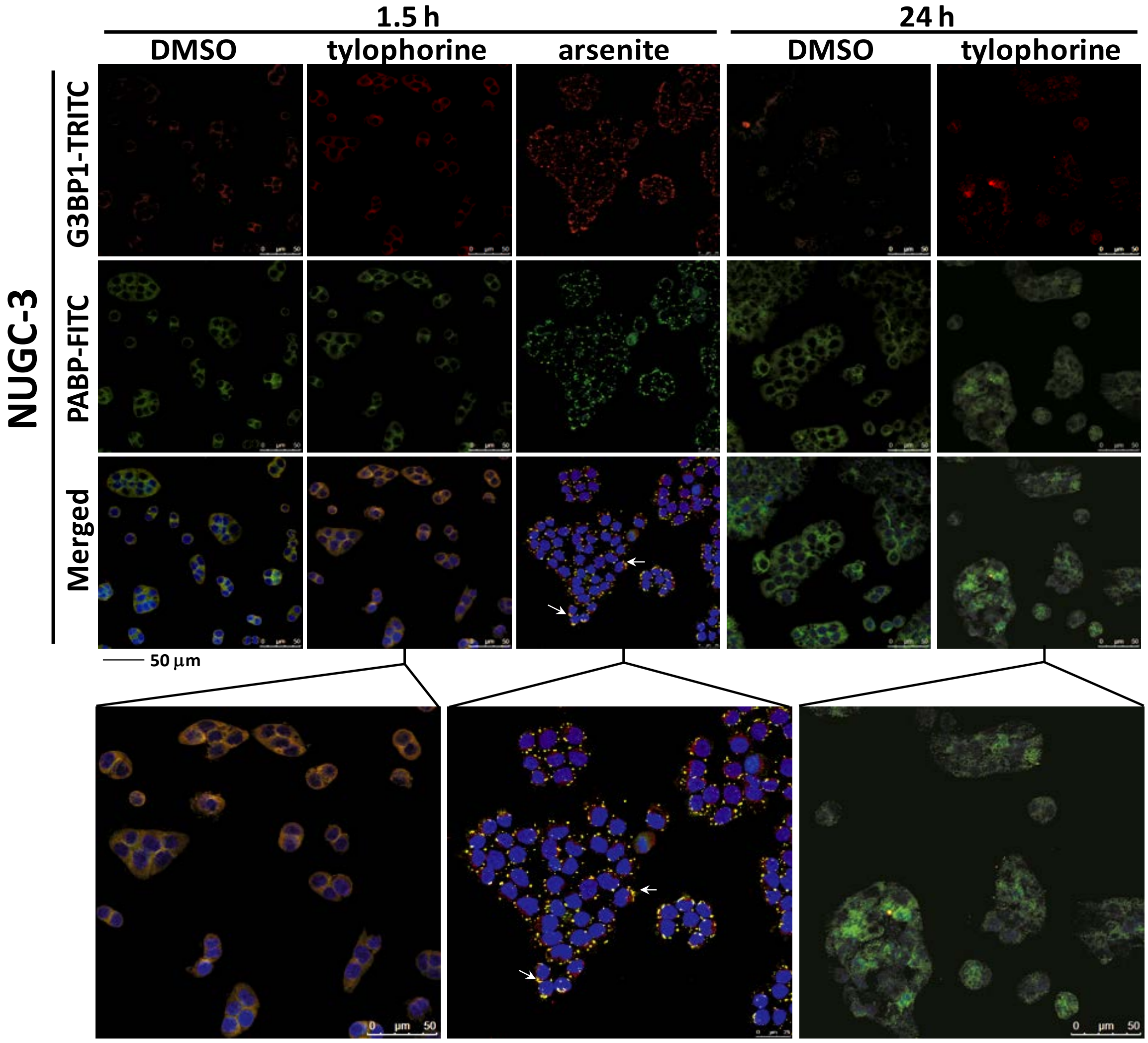

B

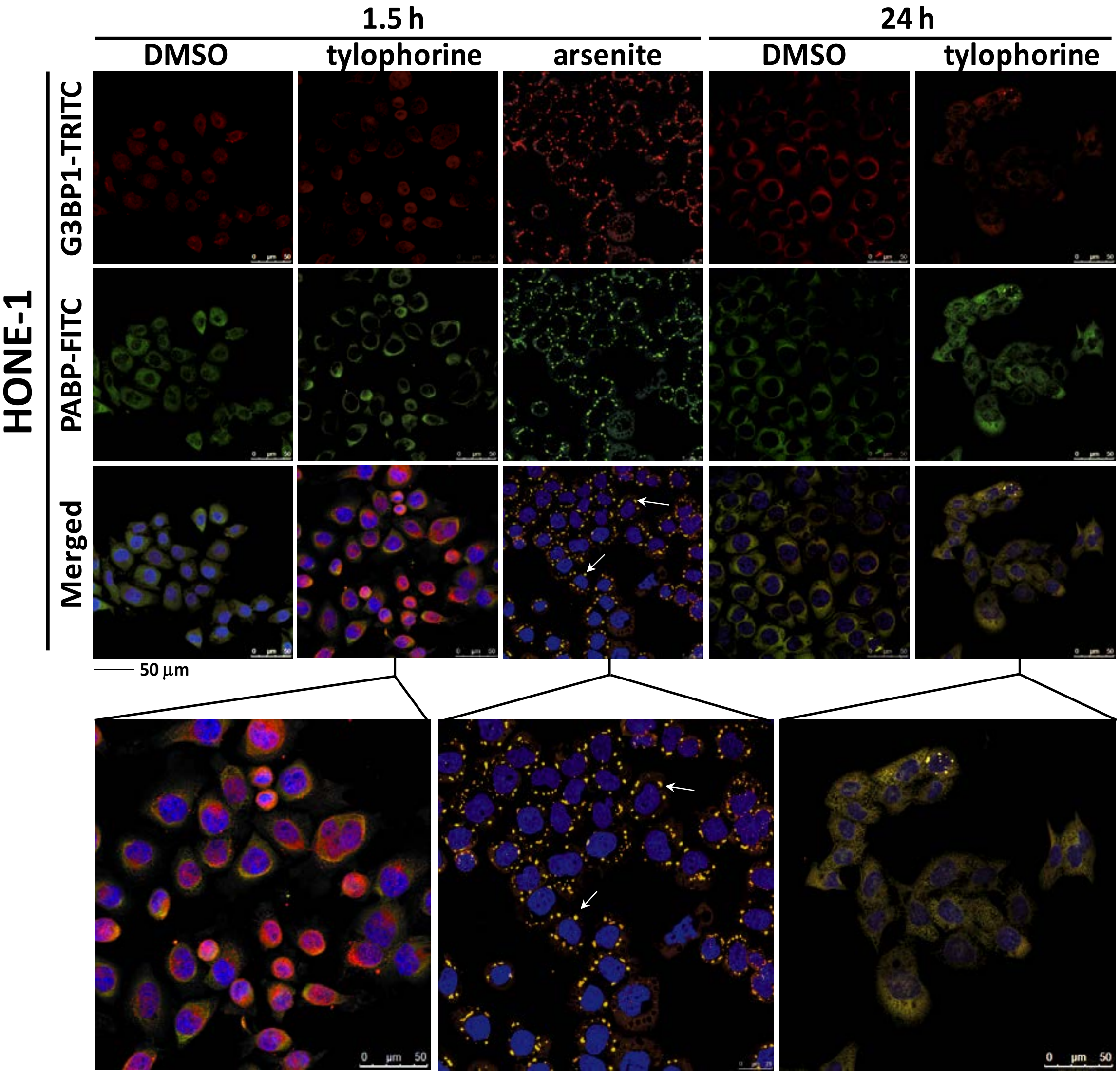
1.5 h
24 h
DMSO
tylophorine
arsenite
DMSO
tylophorine
HONE-1
G3BP1-TRITC
PABP-FITC
Merged
50 μm
0 μm 50

C

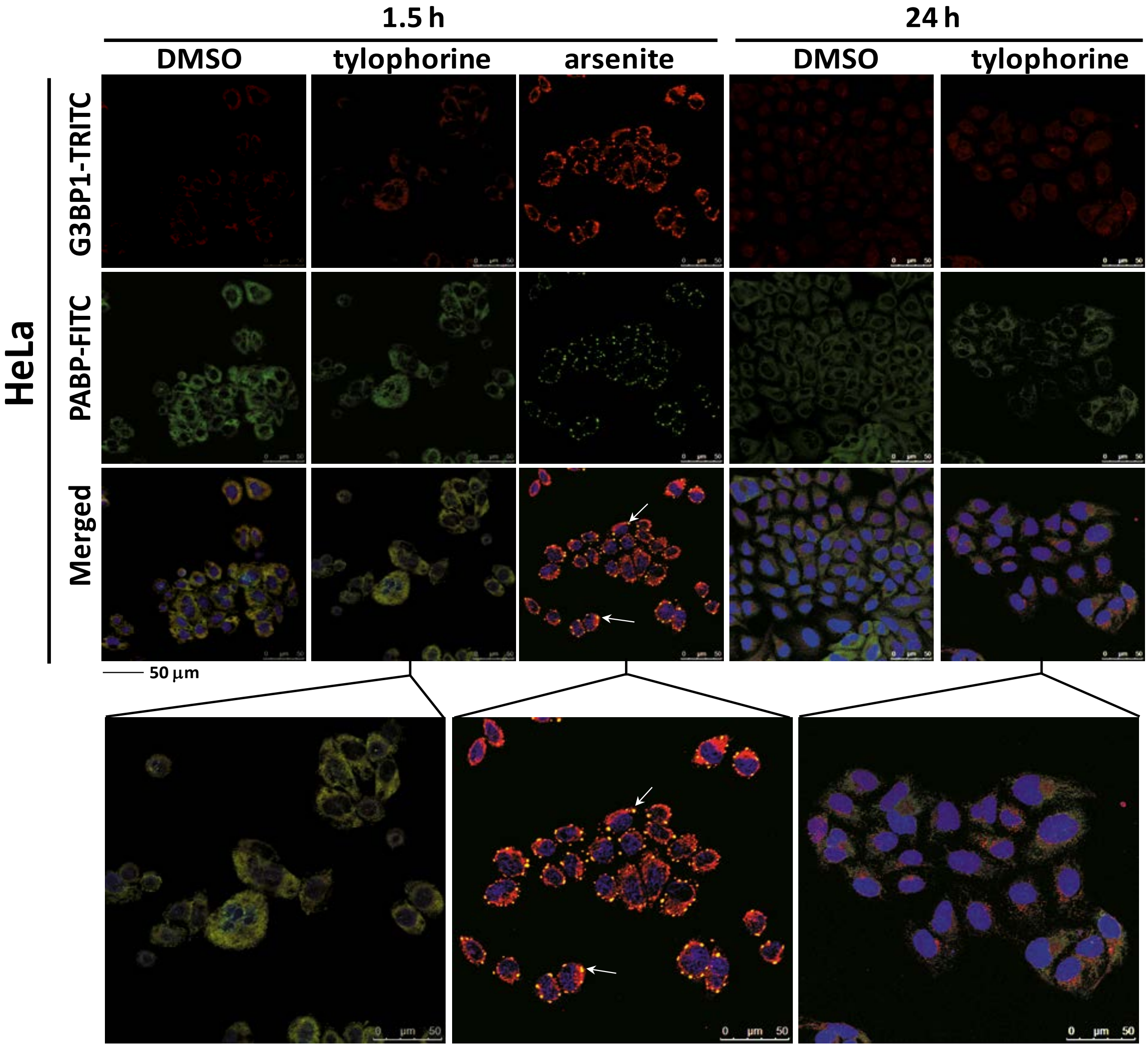

D

	1.5 h			24 h	
	DMSO	tylophorine	arsenite	DMSO	tylophorine
**NUGC-3**	222 (3)*	174 (8)	330 (1426)	356(39)	220(11)
**HONE-1**	161 (5)	162 (8)	152 (1227)	218 (47)	160 (44)
**Hela**	195 (20)	176 (28)	213 (1400)	229(17)	201(52)

*** total stress granules number present (total cell number counted)**

8. **Supplemental Figure 2. Tylophorine treatment significantly decreased the PTEN protein levels in HONE-1, NUGC-3, and MCF7 carcinoma cells.** Cells were treated with vehicle DMSO or tylophorine for 24 h. The resultant cell lysates were analyzed by western blotting with the antibodies indicated. The results shown are representative of 3 independent experiments.

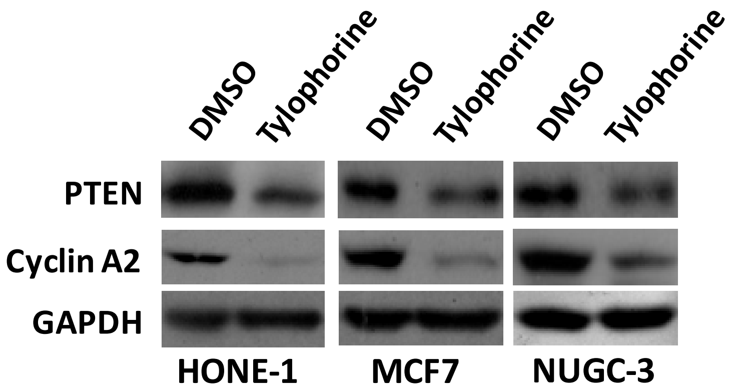

9. **Supplemental Figure 3. Ectopically overexpressed c-Myc did not affect the c-Jun accumulation by tylophorine.** HONE-1 cells were transfected with expression vectors indicated for 24 h prior to vehicle DMSO or tylophorine treatment as indicated concentrations for another 24 h. The resultant cell lysates were analyzed by western blotting with the antibodies indicated. The results shown are representative of 3 independent experiments.

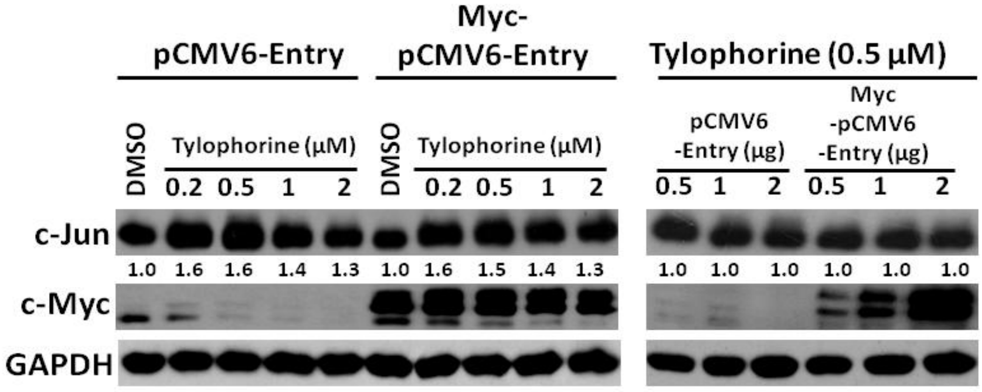

10. **Supplemental Figure 4.** ^{1}H-NMR and ^{13}C-NMR data of biotinylated tylophorine (BT).

^{1}H-NMR spectrum

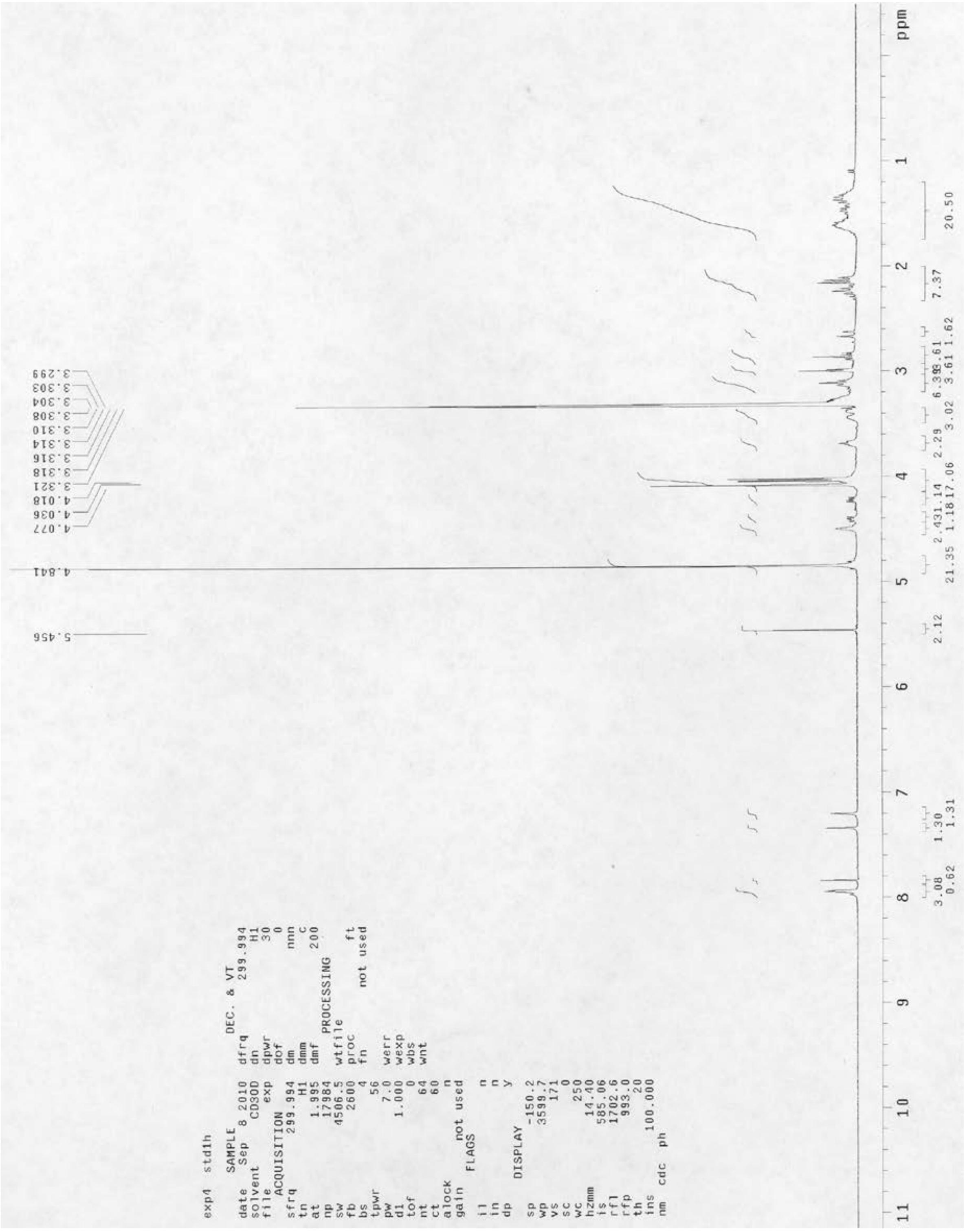

^{13}C-NMR spectrum

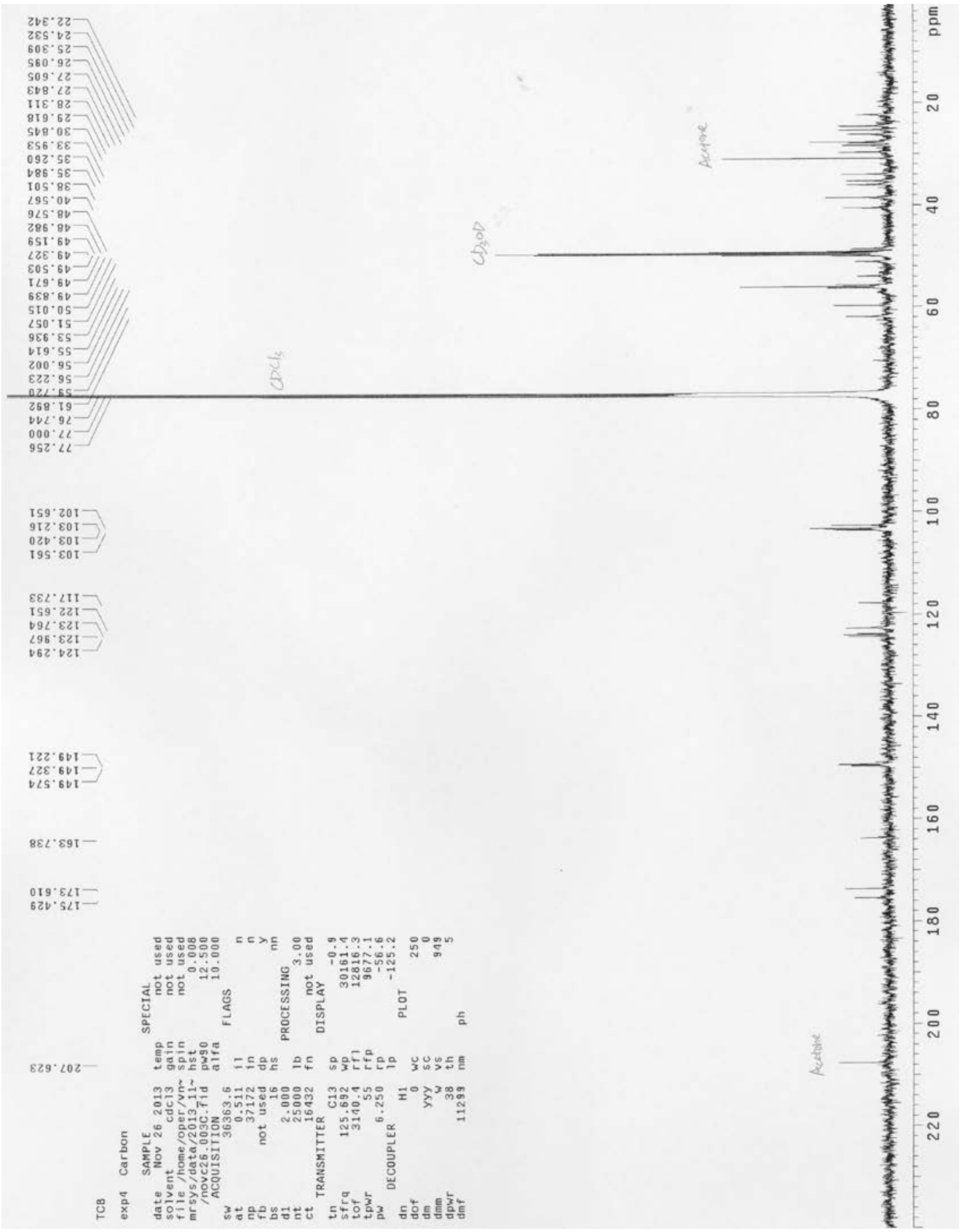

Supplement: Supplementary file 1 [file oncotarget-06-2148-s001.pdf]
